# Comparable Toxicity of Surface-Modified TiO_2_ Nanoparticles: An In Vivo Experimental Study on Reproductive Toxicity in Rats

**DOI:** 10.3390/antiox13020231

**Published:** 2024-02-13

**Authors:** Ana Todorović, Katarina Bobić, Filip Veljković, Snežana Pejić, Sofija Glumac, Sanja Stanković, Tijana Milovanović, Ivana Vukoje, Jovan M. Nedeljković, Sanja Radojević Škodrić, Snežana B. Pajović, Dunja Drakulić

**Affiliations:** 1 Department of Molecular Biology and Endocrinology, VINČA Institute of Nuclear Sciences—National Institute of the Republic of Serbia, University of Belgrade, 11000 Belgrade, Serbia; anato@vin.bg.ac.rs (A.T.); katarina.bobic@vin.bg.ac.rs (K.B.); snezana@vin.bg.ac.rs (S.P.); pajovic@vin.bg.ac.rs (S.B.P.); drakulic@vin.bg.ac.rs (D.D.); 2 Department of Physical Chemistry, VINČA Institute of Nuclear Sciences—National Institute of the Republic of Serbia, University of Belgrade, 11000 Belgrade, Serbia; filipveljkovic@vin.bg.ac.rs; 3 Institute of Pathology, Faculty of Medicine, University of Belgrade, 11000 Belgrade, Serbia; 4 Centre for Medical Biochemistry, University Clinical Centre of Serbia, 11000 Belgrade, Serbia; sanjast2013@gmail.com; 5 Faculty of Medical Sciences, University of Kragujevac, 550601 Kragujevac, Serbia; 6 Public Company “Nuclear Facilities of Serbia”, 11000 Belgrade, Serbia; tijana.milovanovic@nuklearniobjekti.rs; 7 Department of Radiation Chemistry and Physics, VINČA Institute of Nuclear Sciences—National Institute of the Republic of Serbia, University of Belgrade, 11000 Belgrade, Serbia; ivanav@vin.bg.ac.rs (I.V.); jovned@vin.bg.ac.rs (J.M.N.)

**Keywords:** TiO_2_, nanoparticles, chemical modifications, reproductive organs, toxicity, oxidative stress, hormonal status, rats

## Abstract

Nanoparticles (NPs), a distinct class of particles ranging in size from 1 to 100 nm, are one of the most promising technologies of the 21st century, and titanium dioxide NPs (TiO_2_ NPs) are among the most widely produced and used NPs globally. The increased application of TiO_2_ NPs raises concerns regarding their global safety and risks of exposure. Many animal studies have reported the accumulation of TiO_2_ NPs in female reproductive organs; however, evidence of the resultant toxicity remains ambiguous. Since the surface area and chemical modifications of NPs can significantly change their cytotoxicity, we aimed to compare the toxic effects of pristine TiO_2_ powder with surface-modified TiO_2_ powders with salicylic acid (TiO_2_/SA) and 5-aminosalicylic acid (TiO_2_/5-ASA) on the ovaries, oviducts, and uterus on the 14th day following acute oral treatment. The results, based on alterations in food and water intake, body mass, organ-to-body mass ratio, hormonal status, histological features of tissues of interest, and antioxidant parameters, suggest that the modification with 5-ASA can mitigate some of the observed toxic effects of TiO_2_ powder and encourage future investigations to create NPs that can potentially reduce the harmful effects of TiO_2_ NPs while preserving their positive impacts.

## 1. Introduction

Nanoparticles (NPs) are a distinct class of particles ranging in size from 1 to 100 nm, including the surrounding interfacial layer [1]. Concerning all NPs, titanium dioxide NPs (TiO_2_ NPs) are among the most widely produced and used globally [2], with a crucial role in nanopharmaceuticals and nanomedicine [3], cosmetics [4], food [5], and agroindustry [6], experiencing an escalating production with the advancement of nanotechnology.

The intake of TiO_2_ NPs by food consumption commonly occurs through the ingestion of E171 TiO_2_, an additive for enhancing the white color of certain foodstuffs, such as sweets or milk-based products [7], but also with the use of pharmaceuticals [8] and toothpaste [9]. The research findings reveal that the daily intake of E171 reaches several hundred milligrams (mg), with approximately 36% in the nanoscale range [10]. According to Farrell and Magnuson [11], approximately 99% of ingested TiO_2_ NPs remain unchanged and are excreted through the fecal route, but small amounts (not exceeding 0.1%) are absorbed along the oro-gastrointestinal route and distributed to various organs and tissues, including the reproductive system.

The significance of fertility, reproduction, and fetal development underscores the importance of growing public awareness of the detrimental effects of NPs on the reproductive system. This is especially important because NPs, compared to larger particles, exhibit increased solubility and an enhanced capacity to translocate across the intestinal epithelium, potentially leading to harmful side effects [12]. Various animal studies have reported the accumulation of NPs in female reproductive organs; however, evidence of the resultant toxicity remains ambiguous.

Wang et al. (2007) investigated the acute toxicity and biodistribution of differently sized TiO_2_ particles in mice following oral administration and indicated that TiO_2_ particles could be transported to other tissues and organs after uptake by the gastrointestinal tract; however, they did not observe abnormal pathological changes in the heart, lung, testicle (ovary), and spleen tissues [13]. In addition, in a study by Chen et al. (2020), Sprague Dawley rats were orally administered varying doses of TiO_2_ NPs for 90 days. Although limited absorption and distribution levels of TiO_2_ NPs were observed in rat tissues, significant tissue-specific oxidative stress and elemental imbalance were noted. The authors also performed a correlation analysis, revealing the complex connection between elemental imbalances and oxidative stress, emphasizing the potential for subsequent adverse health effects [14].

An examination of the effects of orally administered TiO_2_ NPs on mouse embryonic development indicated histological changes in the ovaries, reduced pregnancy rates, and impaired in vitro fertility. Ovarian dysfunction, characterized by the degeneration of follicles and cyst formation, was accompanied by elevated lipid peroxidation end products and estrogen hormone levels, emphasizing its adverse impact on reproductive health [15]. Histopathological changes in the reproductive organs and spleen with altered serum hormone levels, including testosterone and 17-β-estradiol, were also detected in a study investigating the impact of short-term oral exposure to anatase TiO_2_ NPs on Sprague Dawley rats [16]. In a long-term exposure study conducted by Gao et al. (2012), female mice subjected to an intragastric administration of TiO_2_ NPs exhibited ovarian injury, imbalance in mineral element distribution, and altered sex hormone levels. Microarray analysis identified differential gene expression in the ovaries, pointing towards potential molecular mechanisms underlying TiO_2_ NP-induced ovarian dysfunction and reduced fertility [17].

Some data indicate that the detrimental effects of NPs on the reproductive system caused by toxicity due to the generation of reactive oxygen species (ROS) can be significantly altered by the surface modification of NPs [18]. The surface modification of TiO_2_ NPs with salicylic acid (SA) and its derivatives leads to the formation of covalent Ti–O–C linkages between the inorganic and organic components of the hybrid [19]. Owing to the interfacial charge transfer (ICT) complex formation, the obtained hybrids displayed absorption activity in the visible spectral range with an enhanced ability to induce photo-driven catalytic reactions, including antimicrobial action [20]. However, the literature concerning the biological impact of ICT complexes is rare, almost non-existent, and includes a limited number of in vitro [21,22] and in vivo [23] studies.

In consideration of all the previously discussed studies, our study aims to assess and compare the potentially toxic effects of acute oral treatment with pristine TiO_2_ powder, and TiO_2_ powders surface-modified with salicylic acid (TiO_2_/SA) or 5-aminosalicylic acid (TiO_2_/5-ASA). In addition to highlighting the potential reproductive toxicity associated with TiO_2_ NPs, the novelty of our research is the examination of surface modifications as a strategy to enhance the safety profile of these NPs. This is achieved through a comprehensive comparative analysis of pristine and surface-modified TiO_2_, covering multiple levels, including food and water intake, body mass and organ-to-body mass ratio, regularity of the estrous cycle, hormonal status, tumor markers, histological features, and antioxidant parameters in rat ovaries, oviducts, and uterus. The future aim of our research involves designing and exploring less toxic NPs, such as TiO_2_/5-ASA, with the objective of understanding their toxicity mechanisms to enable safer applications in response to the increasing importance of balancing the advantages of TiO_2_ NP use with the imperative to minimize their harm.

## 2. Materials and Methods

### 2.1. Chemicals

Analytical-grade salts, buffer reagents, chloramine T, hydrochloric acid (HCl), acetic acid (CH_3_COOH), potassium iodide (KI), methanesulfonic acid (CH_4_O_3_S), adrenaline, glutathione reductase (GR), peroxidase, and ethylenediaminetetraacetic acid (EDTA) were purchased from Sigma-Aldrich, St. Louis, MO, USA. 3,3′,5,5′-tetramethylbenzidine (TMB), nicotine adenine dinucleotide phosphate (NADPH), L glutathione reduced (GSH), and oxidized (GSSG) were from SERVA Electrophoresis GmbH, Heidelberg, Germany. Hydrogen peroxide (H_2_O_2_), methanol, and 2,5-dihydroxybenzoic acid (DHB) were provided by Merck KGaA, Darmstadt, Germany. 5,5′-dithio-bis-2-nitrobenzoic acid (DTNB) was purchased from Acros Organics, Geel, Belgium, while 1-methyl-2-phenylindole (MFI) and uric acid were from Alfa Aesar, Ward Hill, MA, USA. Dimethyl sulfoxide (DMSO), chloroform, and acetonitrile (C_2_H_3_N) were from Fisher Scientific, Waltham, MA, USA.

### 2.2. Synthesis of Surface-Modified TiO_2_ NPs with SA and 5-ASA

The surface-modified TiO_2_ powders (Degussa P25, Sigma Aldrich, St. Louis, MO, USA) with SA and 5-ASA were prepared by taking advantage of the condensation reaction of hydroxyl groups from the inorganic and organic components of the hybrid, as described elsewhere [24,25]. Briefly, 3.5 g of TiO_2_ powder was combined with 602 mg of SA (or 667 mg of 5-ASA) in 100 mL of water. The dispersion was stirred for 24 h at 40 °C. Then, the powder was separated by centrifugation, washed three times with deionized water to remove excess ligands, and finally dried in the vacuum oven at 40 °C. The coloration of the powders indicated the formation of inorganic–organic hybrids (TiO_2_/SA and TiO_2_/5-ASA).

### 2.3. Optical Characterization of Surface-Modified TiO_2_ NPs with SA and 5-ASA

Diffuse reflection spectra of TiO_2_ NP powders, pristine and surface-modified, were measured using UV-Visible UV-2600 spectrophotometer (Shimadzu, Tokyo, Japan) equipped with an integrated sphere (ISR—2600 Plus). Transmission electron microscopy (TEM) was performed using a JEM-2100 LaB6 instrument (JEOL, Tokyo, Japan) operated at 200 kV. TEM images (Figure S1) were acquired with an Orius CCD camera (Gatan Inc, Pleasanton, CA, USA) at a 2× binning rate.

### 2.4. Animals and Experimental Design

All research procedures were in agreement with the Directive 2010/63/EU of the European Parliament and were authorized by the Veterinary Directorate of the Ministry of Agriculture, Forestry, and Water Management of the Republic of Serbia, with license number 323-07-03626/2021-05. Appropriate measures were implemented to minimize the number of animals utilized and to alleviate their pain and discomfort.

For experimental purposes, young, adult, female Wistar rats with regular estrous cycles and an average body mass of 296.53 ± 2.62 g were used. The animals were housed 2–3 rats per cage, under consistent environmental conditions, consisting of a 12 h light/dark cycle, ambient temperature of 22 ± 2 °C, and relative humidity of 55%. Additionally, they had unrestricted access to standard rat food (Veterinary Institute, Subotica, Republic of Serbia) and tap water. On the first day of the experiment, the rats were fasted for 4 h and randomly assigned to 4 groups, with each group comprising 9 animals. The groups included animals treated with vehicle (0.01 M HCl—a solution used to dissolve NPs) (C group); animals treated with (dispersed) pristine TiO_2_ powder (TiO_2_ group); animals treated with (dispersed) surface-modified TiO_2_ powder with SA (TiO_2_/SA group); and animals treated with (dispersed) surface-modified TiO_2_ powder with 5-ASA (TiO_2_/5-ASA group). All treatments were applied in a non-ovulatory stage of the estrus cycle. Food and water were reintroduced to the animals 2 h following the treatments. All treatments were administered acutely at a dose of 1000 mg/kg by oral gavage using a reusable stainless-steel feeding needle, 16-G4″, with a 3 mm ball diameter (Cadence Inc., Staunton, VA, USA). The dose and application regime were chosen according to the previous toxicological data on the related substances (TiO_2_ NPs and surface-modified TiO_2_ NPs) as described previously [23].

The animals’ general health status, including food and water intake, was systematically monitored daily over a period of 14 days. The individual body mass was evaluated just before treatment (day 0), and on the following test days, 1, 2, 4, 7, 10, and 14, as previously described [23]. Vaginal smear/cytology evaluations were conducted daily by applying a small quantity of vaginal cell suspension to a glass slide and examining it immediately under a light microscope at a 200× magnification rate (Carl Zeiss AG, Oberkocher, Germany).

### 2.5. Tissue Sampling

Fourteen days following the treatments, the rats were quickly decapitated with a guillotine (Harvard Apparatus, Holliston, MA, USA), trunk blood was collected, centrifuged at 3500 rpm for 15 min in Megafuge 2.0R (Heraeus, Hanau, Germany), and the obtained serum was stored at −80 °C until the analyses. The ovaries, oviducts, and uteri of all animals were isolated and weighed, with the ratio of wet tissue mass (mg) to body mass (g) used to express the fractional contributions of these organs to the overall body mass. The reproductive organs of 5 animals per group were stored at −80 °C for further homogenizing, while the tissues of the other 4 animals per group were prepared in a fixation solution (10% buffered formaldehyde) for subsequent histological analyses.

The tissue samples were homogenized with IKA T 10 Basic Ultra Turrax Homogenizer (IKA^®^-Werke GmbH & Co. KG, Staufen, Germany) in cold phosphate-buffered saline (PBS) (1:4 mass/volume ratio) and centrifuged at 13,000× *g* for 30 min at 4 °C in Microcentrifuge 5417R (Eppendorf, Hamburg, Germany). The resulting supernatants were collected and stored at −80 °C until further analysis.

Paraffin sections, approximately 5 μm thick and cut using a rotatory microtome (Leica, Wetzlar, Germany), were stained with hematoxylin and eosin (Sigma-Aldrich, St. Louis, MO, USA). Histopathological changes were estimated using an BX43 microscope (Olympus, Tokyo, Japan) equipped with a digital camera Leica ICC50W (Leica, Wetzlar, Germany) and Olympus DP-SOFT 5.0 program (Olympus, Tokyo, Japan) for photo documentation.

### 2.6. Biochemical Analysis

#### 2.6.1. Serum Sex Hormones and Tumor Markers Analyses

Serum levels of sex hormones (estrogen (E), progesterone (P4), testosterone (T), sex hormone-binding globulin (SHBG), follicle-stimulating hormone (FSH), luteinizing hormone (LH), and prolactin (PRL)), as well as tumor markers (human epididymis protein 4 (HE4) and carcinoma antigen 125 (CA-125)), were measured by electrochemiluminescence immunoassays, using the Roche Cobas e601 automated analyzer (Roche Diagnostics GmbH, Penzberg, Germany).

#### 2.6.2. Tissue Oxidative Stress Biomarkers Analyses

The evaluation of the prooxidant/antioxidant balance (PAB) was performed according to the method of Alamdari and coworkers [26]. The working solution (TMB cation solution + TMB solution) was mixed with sample/standard/blank and incubated in a dark place for 12 min, at 37 °C, after which, the reaction was stopped by adding 2 N of HCl, and the absorbance was measured on a microplate reader (WALLAC 1420-Victor2 Multilabel Counter, PerkinElmer, Inc., Shelton, CT, USA) at 450 nm. PAB values were calculated and expressed in arbitrary units (HKs).

Levels of advanced oxidation protein products (AOPPs) were estimated according to the Witko-Sarsat method [27]. Briefly, samples or chloramine-T as the standard were diluted in phosphate buffer, KI, and CH_3_COOH, and the absorbance was measured on a microplate reader (WALLAC 1420-Victor2 Multilabel Counter, PerkinElmer, Inc., Shelton, CT, USA) at 340 nm. The AOPP level was expressed as μmol/L of chloramine-T equivalents.

The final products of lipid oxidation, malondialdehyde (MDA), and 4-hydroxynonenal (HNE) were determined following the method of Gérard-Monnier and coworkers [28]. A solution of MFI in a mixture of acetonitrile/methanol was added to the sample. For the determination of MDA, the reaction was started by adding HCl while CH_4_O_3_S and FeCl_3_ were used for the determinations of MDA and HNE. The absorbance at 586 nm was measured on a microplate reader (WALLAC 1420-Victor2 Multilabel Counter, PerkinElmer, Inc., Shelton, CT, USA) upon incubation of the reaction mixture at 45 °C for 60 min. MDA and HNE concentrations were determined using the corresponding standard curves and expressed in µM.

Total SOD activity was measured by the method of Misra and Fridovich [29], based on the SOD capability to inhibit the conversion of adrenaline to adrenochrome. Manganese superoxide dismutase (MnSOD) activity was estimated by the same method, after the inhibition of copper–zinc–superoxide dismutase (CuZnSOD) with potassium cyanide. The reaction was monitored at 26 °C on an S-40 Boeco spectrophotometer (Boeco, Hamburg, Germany) at 480 nm. CuZnSOD activity was calculated as a difference between the activities of total SOD and MnSOD. One unit of SOD activity was defined as the amount of enzyme that inhibited 50% of the adrenaline autoxidation. The results are expressed as the specific activity of the enzyme in 1 mg of tissue (U/mg).

CAT activity was assayed by the method of Beutler [30] by measuring the absorbance decrease on an S-40 Boeco spectrophotometer (Boeco, Hamburg, Germany), at 240 nm. A decrease in absorbance was the result of H_2_O_2_ decomposition and was proportional to the enzyme’s activity. One unit of CAT activity was defined as the amount of enzyme causing about 90% destruction of the substrate in 1 min in 1 mL of reaction mixture. CAT activity was expressed as the specific activity of the enzyme in 1 mg of tissue (U/mg).

GPx activity was determined by the assay based on coupling the oxidation of GSH and NADPH using GR [31]. A decline in absorbance at 340 nm caused by the oxidation of NADPH measured on a microplate reader (WALLAC 1420-Victor2 Multilabel Counter, PerkinElmer, Inc., Shelton, CT, USA) was proportional to the GPx activity in the sample and expressed as U/g of tissue.

Levels of GSH and GSSG were determined by the method of Salbitani and coworkers [32]. Samples were added to Na-phosphate buffer containing EDTA and DTNB. The GSH concentration was monitored after 5 min on an S-40 Boeco spectrophotometer (Boeco, Hamburg, Germany), at 412 nm. To determine the content of total glutathione (GSH plus GSSG), NADPH and GR were added to the reaction mixture, and the absorbance was measured after 30 min, at 412 nm. Concentrations of GSH and GSSG were calculated and expressed in µM.

The total lipid extracts were prepared by the modified Folch procedure [33] using a chloroform/methanol/water solvent system. Following the resuspension in 0.5 M of DHB matrix solution, the samples were placed into the wells of the stainless-steel target plate and left to dry. The mass spectra were obtained using the reflector mode and “delayed extraction” conditions (delay time was approximately 100 ns) on a commercial MALDI-TOF (matrix-assisted laser desorption/ionization–time-of-flight) Voyager-DE PRO mass spectrometer (Sciex, Framingham CT, USA). Data Explorer Software version 4.9 (Applied Biosystems, Inc., Foster City, CA, USA) was used for the spectra processing.

### 2.7. Statistical Analyses

The results of the general health status and oxidative stress biomarkers are presented as the mean ± standard error of measurement (SEM) while levels of sex hormones and tumor markers are expressed as the mean ± standard deviation (SD). The Statistical software package Graphpad Prism version 4.0 (GraphPad Software, Inc., La Jolla, CA, USA) was used for all the analyses. Multi-group comparisons of the means were performed by one-way ANOVA followed by Tukey’s post-hoc test. The statistical significance was defined at *p* < 0.05.

## 3. Results and Discussion

The literature suggests that oral exposure is a significant absorption pathway for different NPs, including TiO_2_ NPs, owing to the regular consumption of food products, liquid beverages, and drugs containing NPs. Orally ingested TiO_2_ NPs can be transported through the gastrointestinal tract, enter the bloodstream, and accumulate in secondary organs, thereby inducing structural and functional damage [18,34]. The structure, size, and coating of NPs can affect their surface charge, aggregation, and sedimentation, thus affecting their toxicity to the human body [35]. Therefore, we chose a commercial Degussa P25 TiO_2_ nanopowder whose microstructural characteristics (phase composition, particle size, specific surface area, and porosity) are well described in the literature in detail [36,37]. It is well known that the size of Degussa P25 TiO_2_ particles ranges from 20 to 30 nm, and we confirmed that by the TEM measurements (Supplementary Material Figure S1). The size of TiO_2_ particles is not affected by surface modifications with SA and 5-ASA.

Owing to the toxicity issues of TiO_2_ and the lack of available data concerning the potential acute toxicity of surface-modified TiO_2_ powders with SA and 5-ASA, we aimed to evaluate their outcomes during animal life and at the terminal experimental endpoint. These outcomes encompassed a variety of parameters, such as body mass variations, food and water consumption, regularity of the estrous cycle, hormonal and tumor marker levels, organ weights, histological alterations, and levels of redox parameters in rats’ reproductive organs (ovaries, oviducts, and uterus). The data obtained from our study suggest that, in acute oral treatment, the surface modification of TiO_2_ with 5-ASA can potentially attenuate the observed TiO_2_ toxic effects.

**Figure 1:**
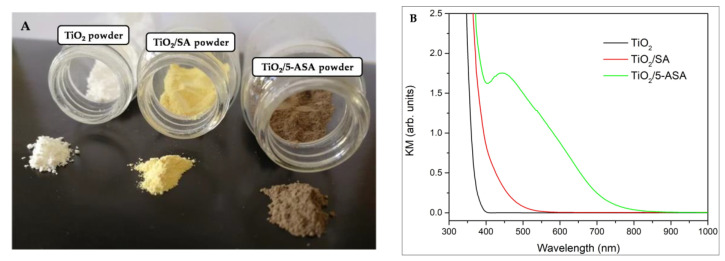
(**A**) The photo images of pristine TiO_2_ powder (white) and inorganic–organic hybrids surface-modified with salicylic acid (TiO_2_/SA) (yellow) and 5-aminosalicylic acid (TiO_2_/5-ASA) (brown). (**B**) The Kubelka–Munk transformations of diffuse reflection data for the unmodified TiO_2_ powder and surface-modified TiO_2_ powders.

Before the oral administration of surface-modified TiO_2_ powders with SA and 5-ASA, we optically characterized the prepared samples to ensure the successful functionalization of TiO_2_. The Kubelka–Munk transformations of the diffuse reflection data of the prepared samples used in the biological tests, including their photo images, are shown in Figure 1. The red absorption shifts upon the surface modification of TiO_2_ with SA and 5-ASA agree with the published literature data, both experimental [24,25] and theoretical [25], calculated using the density functional theory. The difference in the optical response between TiO_2_/SA and TiO_2_/5-ASA was due to the electron-donating nature of the amino groups that remained free after the coordination of 5-ASA to the surface Ti atoms [25].

Alterations in animal body weight, food, and water intake are fundamental parameters for the estimation of toxicity during preclinical studies. These parameters provide valuable information regarding the potential adverse effects of a substance on an organism. Changes in body weight serve as an indicator of systemic toxicity, with changes in food consumption and significant weight loss suggesting adverse effects on various organ systems or metabolic processes. Reduced food intake may indicate the impairment of normal metabolic processes and impact the nutritional status and overall health of animals. Similarly, changes in water intake can provide insights into the potential effects on renal function, whereas dehydration or excessive water consumption can be indicative of specific toxic effects.

The alterations in the average daily changes in rat body weight, food, and water intake after acute oral treatments with TiO_2_, TiO_2_/SA, and TiO_2_/5-ASA are presented in Table 1. Body mass change in the TiO_2_ groups was expressed as a percentage of the body mass alteration in the control group. Moreover, in all TiO_2_ groups, food consumption decreased by 14–16% and water consumption by 11–21% relative to the controls. Compared with the control group, the daily intake of food and water was significantly lower in the groups treated with TiO_2_ and TiO_2_/SA, and the decrease in water consumption was more pronounced in animals exposed to TiO_2_. The detected reductions in food and water intake could be further associated with the observed weight gain stagnation in TiO_2_/SA and TiO_2_/5-ASA animals and with significant body mass loss in rats exposed to TiO_2_. The variations in the listed parameters indicate that TiO_2_ NP-induced toxicity, reflected by body mass loss and decreases in food and water consumption, could be, at least partially, attenuated by surface modifications of pristine TiO_2_ powder with SA and 5-ASA. Similar to our findings, other authors also demonstrated that exposure to zinc oxide (ZnO) or TiO_2_ NPs led to a reduction in body mass [38,39]; however, some experimental results show that the oral intake of TiO_2_ NPs has no significant effect on animal weight [40]. One potential mechanism underlying the impact of NPs on animal weight fluctuations can be associated with the disrupted functional integrity of intestinal epithelial cells [41]. According to these authors, exposure to NPs results in a reduction in absorptive microvilli in intestinal epithelial cells and disrupts their normal structure, which is responsible for nutrient absorption. Additionally, the altered gene expression of nutrient transporter proteins indicates that cells actively respond to the disturbance caused by NP ingestion by attempting to regulate the affected transport mechanisms. Li and coworkers [42] showed that environmental NPs, including TiO_2_, could have a destructive effect on intestinal microbial homeostasis, which could also be one of the potential causes for the observed changes in weight, but also in food and water intake.

Females are particularly vulnerable to NP toxicity. In vitro and in vivo studies indicated that both short- and long-term exposure to low- and high-dose NPs can have detrimental effects on female reproductive organs and normal reproductive functions [18]. For instance, an in vitro study on granulosa rat ovarian cells reported that NPs could penetrate cells and their subcellular organelles, modifying their normal function, including estrogen secretion [43], which could later induce ovum dysplasia. Female in vivo reproductive NP toxicity studies generally focus on the effects of NPs on reproductive ability, prenatal effects, and the impacts on offspring during the perinatal period [44], since total NP tissue levels are increased in female reproductive organs as well as in the fetus [18]. Tassinari et al. [16] reported the accumulation of titanium (Ti) in the ovaries of young, sexually mature rats following short-term oral TiO_2_ NP treatments, as well as a dose-related increase in the number of ovarian apoptotic-like granulosa cells. Long-term oral TiO_2_ exposure in adult mice was shown to induce ovarian damage reflected by inflammation and follicular atresia, along with biochemical dysfunction, sex hormone imbalance, alterations in ovarian-related gene expression, and reduced fertility or pregnancy rate [15,17]. The results of other in vivo studies also show that exposure to TiO_2_ can lead to an imbalance of sex hormones and autoimmunity markers [45], the disturbance of steroidogenesis, which results in a reduction in fertility and follicle development [46], histological alterations in the ovary, including ovarian cyst formation [15], and impact on cytoskeleton arrangement and transzonal projections (TZPs) between somatic cells and oocytes, which play a central role in the fine regulation of normal oocyte and follicle development [47]. Additionally, it was shown that exposure to TiO_2_ NPs affected the expression levels of 288 genes involved in hormone and cytokine pathways in a mouse ovary [17].

**Figure 2:**
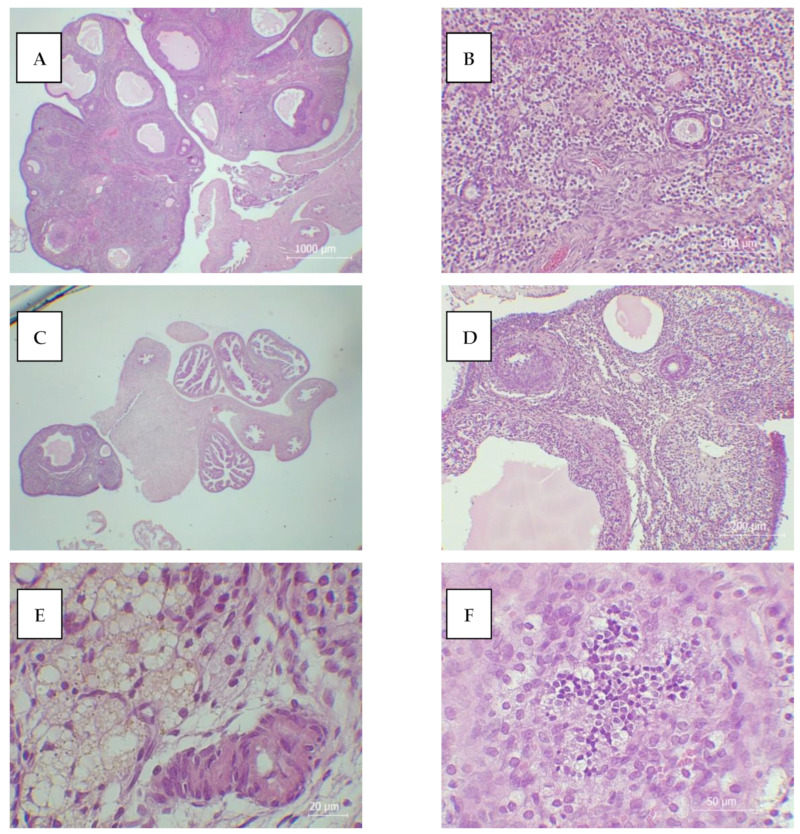
The histological analysis of the ovary samples from the control animals, normal development of primary follicle (**A**) and secondary follicle (**B**), and from the TiO_2_ and TiO_2_/SA groups, ovarian atrophy with no distinct ovarian follicle (**C**), disturbance of primary and second follicle developments, with no oocyte inside (**D**), and TiO_2_ deposition in ovarian tissue (**E**) with inflammatory cell infiltration, apoptosis, and necrosis (**F**).

Our results, presented in Figure 2 and Table 2 and Table 3, depict the histological changes in the ovaries and fractional contribution of the ovaries, oviducts, and uterus to the overall body mass, hormone status, and tumor marker levels 14 days after acute oral applications of TiO_2_ NPs, TiO_2_/SA, and TiO_2_/5-ASA. Before the treatments, all 36 rats had regular estrus cycles of 4 days, synchronized within cages, and generally, a uniform distribution of the different estrous phases/days. Following the vehicle treatment, the animals retained a normal estrus cycle lasting 4 days with a normal development of primary and secondary follicles (Figure 2A,B).

Animals in the TiO_2_- and TiO_2_/SA-treated groups had proestrus as the dominant phase, along with elevated serum estrogen and decreased progesterone levels (Table 3). These results are in accordance with previous studies that reported that TiO_2_ NPs induced sex steroid imbalance, irregular estrus periods, and lower mating and pregnancy rates [15,17]. Moreover, our findings indicate that the ovarian endocrine components of TiO_2_- and TiO_2_/SA-treated animals are targets of NPs, as evidenced by the increase in abnormal (apoptosis and necrosis-like) morphologies of secretory cells, large atretic follicles, severe inflammatory cell infiltration, lymphocytosis, and vascular dilation and congestion (Figure 2C–F). The observed ovarian injuries with irregular cycles and significantly higher fractional contributions of ovaries in the TiO_2_ and TiO_2_/SA groups could be associated with the structural damage of the mitochondria and nuclei of ovarian cells, including mitochondrial swelling, rupture, chromatin condensation, and irregularity in the nuclear membrane, as previously reported by Wang et al. [48], but also with modifications in the expression of genes related to estrogen and progesterone synthesis and metabolism [49,50]. It was found that the application of TiO_2_ NPs upregulated Cyp17a1, which is responsible for enhanced estradiol production [51], which is important considering that the expression levels of estradiol and progesterone, secreted by granulosa and luteal cells, are typically used to estimate ovarian endocrine function [52]. In contrast to the TiO_2_ and TiO_2_/SA groups, TiO_2_/5-ASA-treated animals had insignificantly changed fractional contributions of ovaries compared to the controls, and no abnormal ovarian pathological changes, while their estrogen and testosterone levels were increased and progesterone levels were decreased 14 days following the treatment (Table 2 and Table 3). The observed upregulation of estrogen upon TiO_2_/5-ASA treatment could be associated with the increased testosterone level, which was converted into estrogen via aromatase. In addition to estrogen, testosterone could be associated with the direct response of reproductive organs to the effects of NPs [16], displaying an anti-inflammatory capacity by preventing the overexpression of proinflammatory cytokines and creating a tolerogenic immunological milieu in these organs [53]. Considering that, in addition to sex gland secretory hormones, the hypothalamic–pituitary gland axis also plays an important role in hormonal regulation, further research is necessary to clarify the entire scope of the effect of NPs on sex hormones and their underlying mechanisms.

In parallel, in the oviducts and uteri, increased fractional contributions were also observed in all three TiO_2_ NP-treated groups; however, this augmentation was not significant in the group treated with TiO_2_/5-ASA (Table 2). Increased uterine weight with delayed body weight gain was also observed in a pregnant mouse model, treated with cadmium oxide (CdO) NPs [54], although there were various findings where a reduction in uterine mass was detected following the administration of NPs. One such study was by Yamashita et al. [55], who showed that, when compared to the control, pregnant mice treated with 35 nm of TiO_2_ had a decreased body mass and 30% lower uterine weights. Moreover, similar to our observations of TiO_2_ NPs, these authors noticed that modifying nanosilica particles’ (nSPs) surface with –COOH or –NH_2_ functional groups not only prevented alterations in body and uterus weights, but also suppressed oxidative stress and the initiation of the coagulation pathway. The observed alterations are explained by the evidence that NPs in the body are typically coated with serum proteins, triggering diverse cellular responses through protein binding [55]. As different surface characteristics, such as surface charge, are known to affect the binding affinities of proteins to NPs, variations in protein-binding activities between bear and surface-modified NPs could have been responsible for the differences in the toxicity of nanomaterials observed in our experimental setup.

Although the exact molecular mechanisms involved in NPs’ reproductive toxicity are not fully understood and involve apoptosis, inflammation, genotoxicity, and perturbed hormone synthesis [46], oxidative stress is considered the most significant contributor [56,57]. Owing to their strong oxidation potential, excess ROS production induced by NPs can damage biomolecules and cell structures, leading to DNA/RNA impairment, protein and lipid peroxidation, membrane disruption, and consequently cell necrosis and apoptosis. ROS can additionally enhance pro-inflammatory cytokine production and activate inflammatory cells, which further increases ROS production [58]. Studies on polystyrene NPs have shown that NPs can alter the redox system in mouse serum and induce oxidative stress damage to the ovary. The outcomes of these experiments demonstrate significant decreases in the circulating antioxidant markers GSH, CAT, and total antioxidant capacity, as well as an increase in the oxidation product MDA [59]. Hu and coworkers [60] observed that female rat exposure to high doses of Cu NPs induced uterine damage correlated with the increased MDA level and reduced expression of SOD. Furthermore, changes in oxidative stress were associated with alterations in the uterine expression of 963 genes involved in cell cycle, cell proliferation, vasculature development, cell adhesion, angiogenesis, apoptotic process, stress response, ion channel binding, immune response, inflammatory response, and other processes.

Our results also show that different TiO_2_ NPs induce prominent oxidative stress in rat ovaries, oviducts, and uterus. Despite the changes in the activities of specific antioxidant enzymes (MnSOD, CuSOD, CAT, and GPx) and redox balance parameters (GSH/GSSG and PC/LPC), which were observed in all three reproductive organs following treatments with all three types of TiO_2_ NPs, significant alterations in the total oxidation levels of proteins and lipids (AOPP, MDA, and HNE) and the overall pro-antioxidant balance (PAB) were detected in the oviduct and uterus but not in the ovaries (Table 4, Table 5 and Table 6). This indicates that the antioxidant system in rat ovaries can compensate for NP-induced oxidative stress by regulating the levels of antioxidant enzymes and low-molecular-weight antioxidants, while simultaneously maintaining the overall redox balance at the control level. Conversely, the same dose and type of NPs in the uterus and oviduct led to alterations in both the individual and overall parameters of oxidative stress, suggesting a greater nano-sensitivity of these organs.

With respect to the tested modifications, we observed that, compared to the controls, TiO_2_ induced the uppermost perturbation in oxidative stress parameters, while TiO_2_/5-ASA had the mildest impact. Therefore, it can be assumed that a modification with 5-ASA can significantly mitigate the pro-oxidative effect of the pristine TiO_2_ powder. In addition, considering that, in addition to the lowest redox imbalance, the mildest changes in other physiological and hormonal parameters were detected in the TiO_2_/5-ASA group, it could be indirectly concluded that this alignment of outcomes in our findings supported the hypothesis that oxidative stress was the primary mechanism of nanotoxicity.

## 4. Conclusions

Despite the complexity of the nanotoxicity mechanism, which involves the up/downregulation of several hundred genes related to the cell cycle, cell proliferation, vasculature development, cell adhesion, angiogenesis, apoptotic process, stress response, immune response, inflammatory response, and other processes [60], it is apparent that the final response in cells is influenced by the physicochemical characteristics of NPs, such as the shape, size, charge, surface modification, and agglomeration/aggregation [61]. Accordingly, our findings show that the modification with 5-ASA can alleviate the negative impact of the acute oral administration of TiO_2_ on various biochemical, antioxidant, and physiological parameters in rat ovaries, oviducts, and uterus. This is not surprising, given that 5-ASA is a non-steroidal anti-inflammatory drug commonly employed in the treatment of mild to moderate inflammatory bowel disease, effective in capturing free radicals, and recognized as an antioxidant [62]. It is suggested that 5-ASA functions by activating a group of nuclear receptors responsible for regulating processes such as inflammation, cell growth, programmed cell death, and metabolic activities [63], which could explain the diverse range of protective effects observed when 5-ASA is bound to TiO_2_ NPs. However, neither the SA nor 5-ASA single treatment resulted in changes in any of the parameters tested (Supplementary Material Tables S1–S6). Future research can further explore the molecular mechanisms behind this mitigation, assess the long-term effects, and investigate the broader applications in the food, agriculture, and nanopharmaceutical industries. Standardized testing protocols, exposure limits, and environmental impact assessments for these modified nanoparticles should also be the focus of future studies aimed at understanding and utilizing the benefits of modified TiO_2_ NPs in minimizing toxicity and developing safer nanomaterials.

## Figures and Tables

**Table 1:** Average daily intake levels of food (g/day) and water (ml/day) and total body mass gain/loss (g) of young, female rats after the oral administration of pristine TiO_2_ powder and TiO_2_ powders surface-modified with salicylic (SA) and aminosalicylic acid (ASA). C group—animals treated with vehicle (0.01 M HCl—a solution used to dissolve NPs); TiO_2_ group—animals treated with (dispersed) pristine TiO_2_ NPs; TiO_2_/SA group—animals treated with (dispersed) surface-modified TiO_2_ with SA; TiO_2_/5-ASA group—animals treated with (dispersed) surface-modified TiO_2_ with 5-ASA.

Group/Parameter	C	TiO_2_	TiO_2_/SA	TiO_2_/5-ASA
Food intake (g/day)	22.16 ± 0.42 ^a^	18.59 ± 0.41 ^b^	18.59 ± 0.90 ^b^	19.16 ± 0.42 ^b^
Water intake (ml/day)	47.59 ± 0.85 ^a^	37.61 ± 0.44 ^b^	42.16 ± 0.76 ^c^	42.06 ± 0.59 ^c^
Body mass gain/loss (g)	18.89 ± 2.47 ^a^	−10.00 ± 3.23 ^b^	6.11 ± 3.98 ^a,b^	8.33 ± 2.36 ^a,b^

Values with the same letter (a, b, c) did not differ significantly from each other (*p* > 0.05).

**Table 2:** Fractional contribution of ovaries, oviducts, and uterus of young, female rats 14 days after the oral administration of pristine TiO_2_ powder and TiO_2_ powders surface-modified with salicylic (SA) and aminosalicylic acid (ASA). C group—animals treated with vehicle (0.01 M HCl—a solution used to dissolve NPs); TiO_2_ group—animals treated with (dispersed) pristine TiO_2_ NPs; TiO_2_/SA group—animals treated with (dispersed) surface-modified TiO_2_ with SA; TiO_2_/5-ASA group—animals treated with (dispersed) surface-modified TiO_2_ with 5-ASA.

Group/Parameter	C	TiO_2_	TiO_2_/SA	TiO_2_/5-ASA
Body mass (g)	313.89 ± 4.06 ^a^	285.56 ± 1.77 ^b^	303.89 ± 8.85 ^a,b^	306.11 ± 4.98 ^a,b^
Wet ovaries mass/body mass (mg/g)	0.41 ± 0.02 ^a^	0.61 ± 0.06 ^b^	0.73 ± 0.09 ^b^	0.53 ± 0.06 ^a,b^
Wet oviducts and uterus mass/body mass (mg/g)	1.98 ± 0.17 ^a^	5.01 ± 0.42 ^b^	5.03 ± 1.39 ^b^	4.73 ± 0.33 ^a,b^

Values with the same letter (a, b) did not differ significantly from each other (*p* > 0.05).

**Table 3:** Hormone status and tumor marker levels of young, female rats 14 days after the oral administration of pristine TiO_2_ powder and TiO_2_ powders surface-modified with salicylic (SA) and aminosalicylic acid (ASA). C group—animals treated with vehicle (0.01 M HCl—a solution used to dissolve NPs); TiO_2_ group—animals treated with (dispersed) pristine TiO_2_ NPs; TiO_2_/SA group—animals treated with (dispersed) surface-modified TiO_2_ with SA; TiO_2_/5-ASA group—animals treated with (dispersed) surface-modified TiO_2_ with 5-ASA.

Group/Hormone	C	TiO_2_	TiO_2_/SA	TiO_2_/5-ASA
Estradiol (pmol/L)	132.11 ± 23.80 ^a^	169.44 ± 19.93 ^b^	167.44 ± 29.62 ^b^	172.67 ± 27.35 ^b^
Progesterone (nmol/L)	112.77 ± 15.02 ^a^	69.51 ± 25.29 ^b^	37.96 ± 14.43 ^c^	45.48 ± 18.85 ^b,c^
Testosterone (nmol/L)	0.32 ± 0.11 ^a^	0.35 ± 0.09 ^a^	0.33 ± 0.08 ^a^	0.63 ± 0.28 ^b^
Sex hormone-binding globulin (nmol/L)	<4.5	<4.5	<4.5	<4.5
Follicle-stimulating hormone (IU/L)	<0.11	<0.11	<0.11	<0.11
Luteinizing hormone (IU/L)	<0.12	<0.12	<0.12	<0.12
Prolactin (mIU/L)	<17.22	<17.22	<17.22	<17.22
**Group/Tumor Marker**	**C**	**TiO_2_**	**TiO_2_/SA**	**TiO_2_/5-ASA**
HE4 (pmol/L)	<20	<20	<20	<20
CA 125 (IU/mL)	<1.1	<1.1	<1.1	<1.1

Values with the same letter (a, b, c) did not differ significantly from each other (*p* > 0.05).

**Table 4:** Levels of redox markers in ovaries of young, female rats 14 days after the oral administration of pristine TiO_2_ powder and TiO_2_ powders surface-modified with salicylic (SA) and aminosalicylic acid (ASA). C group—animals treated with vehicle (0.01 M HCl—a solution used to dissolve NPs); TiO_2_ group—animals treated with (dispersed) pristine TiO_2_ NPs; TiO_2_/SA group—animals treated with (dispersed) surface-modified TiO_2_ with SA; TiO_2_/5-ASA group—animals treated with (dispersed) surface-modified TiO_2_ with 5-ASA.

Group/Parameter	C	TiO_2_	TiO_2_/SA	TiO_2_/5-ASA
PAB (HKU)	220.22 ± 7.59 ^a^	224.44 ± 6.28 ^a^	229.12 ± 11.47 ^a^	221.92 ± 10.17 ^a^
AOPP (µmol/L)	92.48 ± 7.96 ^a^	108.30 ± 11.36 ^a^	103.48 ± 4.68 ^a^	106.95 ± 10.99 ^a^
MDA (µM)	0.11 ± 0.00 ^a^	0.14 ± 0.01 ^a^	0.21 ± 0.01 ^b^	0.14 ± 0.01 ^a^
HNE (µM)	0.25 ± 0.02 ^a^	0.31 ± 0.01 ^a^	0.49 ± 0.03 ^b^	0.24 ± 0.00 ^a^
MnSOD (U/mg)	4.97 ± 0.58 ^a^	8.45 ± 0.64 ^b^	9.03 ± 0.85 ^b^	6.69 ± 0.73 ^a,b^
CuZnSOD (U/mg)	55.61 ± 5.60 ^a^	34.29 ± 3.07 ^b^	23.96 ± 3.24 ^b^	36.42 ± 3.18 ^b^
CAT (U/mg)	51.10 ± 5.11 ^a^	93.98 ± 6.68 ^b^	20.71 ± 3.73 ^c^	73.47 ± 7.73 ^a,b^
GPx (U/g)	20.03 ± 1.28 ^a^	38.01 ± 1.53 ^b^	9.95 ± 1.47 ^c^	29.07 ± 1.62 ^d^
GSH/GSSG	0.70 ± 0.06 ^a,c^	0.26 ± 0.07 ^b^	0.82 ± 0.14 ^a^	0.36 ± 0.05 ^c,b^
PC/LPC	1.60 ± 0.21 ^a^	0.72 ± 0.02 ^b^	0.87 ± 0.04 ^b^	1.41 ± 0.28 ^a,b^

Values with the same letter (a, b, c) did not differ significantly from each other (*p* > 0.05).

**Table 5:** Levels of redox markers in uteri of young, female rats 14 days after the oral administration of pristine TiO_2_ powder and TiO_2_ powders surface-modified with salicylic (SA) and aminosalicylic acid (ASA). C group—animals treated with vehicle (0.01 M HCl—a solution used to dissolve NPs); TiO_2_ group—animals treated with (dispersed) pristine TiO_2_ NPs; TiO_2_/SA group—animals treated with (dispersed) surface-modified TiO_2_ with SA; TiO_2_/5-ASA group—animals treated with (dispersed) surface-modified TiO_2_ with 5-ASA.

Group/Parameter	C	TiO_2_	TiO_2_/SA	TiO_2_/5-ASA
PAB (HKU)	318.74 ± 10.60 ^a^	374.81 ± 7.78 ^b^	318.23 ± 14.21 ^a^	314.24 ± 10.75 ^a^
AOPP (µmol/L)	62.38 ± 1.53 ^a^	61.06 ± 3.70 ^a^	49.84 ± 2.70 ^b^	44.29 ± 1.67 ^b^
MDA (µM)	0.12 ± 0.01 ^a^	0.19 ± 0.01 ^b^	0.17 ± 0.01 ^b,c^	0.13 ± 0.01 ^a,c^
HNE (µM)	0.25 ± 0.03 ^a^	0.20 ± 0.04 ^a^	0.23 ± 0.04 ^a^	0.38 ± 0.01 ^b^
MnSOD (U/mg)	5.35 ± 0.53 ^a,b^	7.09 ± 0.37 ^a^	6.50 ± 0.66 ^a,b^	4.37 ± 0.75 ^b^
CuZnSOD (U/mg)	36.54 ± 2.66 ^a^	32.61 ± 4.06 ^a^	35.23 ± 3.61 ^a^	30.31 ± 4.72 ^a^
CAT (U/mg)	59.36 ± 4.18 ^a^	75.71 ± 5.71 ^a^	77.37 ± 4.93 ^a^	66.77 ± 3.12 ^a^
GPx (U/g)	33.37 ± 2.74 ^a^	59.91 ± 5.77 ^b^	40.30 ± 3.86 ^a^	38.48 ± 5.25 ^a^
GSH/GSSG	0.87 ± 0.03 ^a^	0.49 ± 0.04 ^b^	0.49 ± 0.05 ^b,c^	0.77 ± 0.13 ^a,c^
PC/LPC	1.59 ± 0.10 ^a,c^	1.12 ± 0.03 ^b^	1.35 ± 0.08 ^b,c^	1.61 ± 0.10 ^c^

Values with the same letter (a, b, c) did not differ significantly from each other (*p* > 0.05).

**Table 6:** Levels of redox markers in oviducts of young, female rats 14 days after the oral administration of pristine TiO_2_ powder and TiO_2_ powders surface-modified with salicylic (SA) and aminosalicylic acid (ASA). C group—animals treated with vehicle (0.01 M HCl—a solution used to dissolve NPs); TiO_2_ group—animals treated with (dispersed) pristine TiO2 NPs; TiO_2_/SA group—animals treated with (dispersed) surface-modified TiO_2_ with SA; TiO_2_/5-ASA group—animals treated with (dispersed) surface-modified TiO_2_ with 5-ASA.

Group/Parameter	C	TiO_2_	TiO_2_/SA	TiO_2_/5-ASA
PAB (HKU)	148.72 ± 11.63 ^a^	225.10 ± 7.39 ^b^	312.90 ± 28.44 ^c^	162.12 ± 14.23 ^a,b^
AOPP (µmol/L)	65.19 ± 4.65 ^a,c^	105.80 ± 8.45 ^a^	161.78 ± 11.18 ^b^	69.36 ± 3.83 ^c^
MDA (µM)	0.15 ± 0.01 ^a,c^	0.21 ± 0.02 ^b^	0.12 ± 0.01 ^a^	0.16 ± 0.01 ^c^
HNE (µM)	0.24 ± 0.04 ^a^	0.20 ± 0.04 ^a^	0.31 ± 0.01 ^a^	0.36 ± 0.04 ^a^
MnSOD (U/mg)	5.00 ± 0.67 ^a^	8.12 ± 0.56 ^b^	6.48 ± 0.71 ^a,b^	5.46 ± 0.89 ^a,b^
CuZnSOD (U/mg)	26.79 ± 2.71 ^a^	51.81 ± 5.56 ^b^	44.64 ± 4.94 ^b^	25.98 ± 3.74 ^a^
CAT (U/mg)	43.30 ± 4.04 ^a,b^	36.15 ± 2.78 ^a^	58.66 ± 6.69 ^b^	33.20 ± 5.00 ^a^
GPx (U/g)	23.80 ± 2.87 ^a^	17.66 ± 2.30 ^a^	24.40 ± 1.14 ^a^	16.41 ± 2.70 ^a^
GSH/GSSG	1.49 ± 0.10 ^a^	0.88 ± 0.05 ^b,c^	0.52 ± 0.09 ^c^	1.07 ± 0.16 ^a,b^
PC/LPC	1.45 ± 0.16 ^a^	1.27 ± 0.13 ^a^	0.74 ± 0.04 ^b^	1.36 ± 0.06 ^a^

Values with the same letter (a, b, c) did not differ significantly from each other (*p* > 0.05).

## Data Availability

All study data are included in the article.

## Supplementary Materials

The following supporting information can be downloaded at: https://www.mdpi.com/article/10.3390/antiox13020231/s1. Figure S1. Typical TEM image of commercial TiO2 nanoparticles (Degussa P25); Table S1. Average daily intake of food (g/day)and water (ml/day) and total body mass gain/loss (g) of young female rats after oral administration of vehicle, SA and 5-ASA treatment. C group—animals treated with vehicle (0.01 M HCl—a solution used to disperse NPs); SA group—animals treated with salicylic acid (SA); 5-ASA group—animals treated with 5-amino salicylic acid (5-ASA); Table S2. Fractional contribution of ovaries, oviducts and uterus of young female rats 14 days after oral administration of of vehicle, SA and 5-ASA treatment. C group—animals treated with vehicle (0.01 M HCl—a solution used to disperse NPs); SA group—animals treated with salicylic acid (SA); 5-ASA group—animals treated with 5-amino salicylic acid (5-ASA); Table S3. Hormone status and tumor marker levels of young female rats 14 days after oral administration of vehicle, SA and 5-ASA treatment. C group—animals treated with vehicle (0.01 M HCl—a solution used to disperse NPs); SA group—animals treated with salicylic acid (SA); 5-ASA group—animals treated with 5-amino salicylic acid (5-ASA); Table S4. Levels of redox markers in ovaries of young female rats 14 days after oral administration of vehicle, SA and 5-ASA treatment. C group—animals treated with vehicle (0.01 M HCl—a solution used to disperse NPs); SA group—animals treated with salicylic acid (SA); 5-ASA group—animals treated with 5-amino salicylic acid (5-ASA); Table S5. Levels of redox markers in uteri of young female rats 14 days after oral administration of vehicle, SA and 5-ASA treatment. C group—animals treated with vehicle (0.01 M HCl—a solution used to disperse NPs); SA group—animals treated with salicylic acid (SA); 5-ASA group—animals treated with 5-amino salicylic acid (5-ASA); Table S6. Levels of redox markers in oviducts of young female rats 14 days after oral administration of vehicle, SA and 5-ASA treatment. C group—animals treated with vehicle (0.01 M HCl—a solution used to dissolve NPs); SA group—animals treated with salicylic acid (SA); 5-ASA group—animals treated with 5-amino salicylic acid (5-ASA).
