# Supplementary material for: Comparable Toxicity of Surface-Modified TiO2 Nanoparticles: An In Vivo Experimental Study on Reproductive Toxicity in Rats
Source: Antioxidants (Basel). 2024 Feb 13;13(2):231. doi: 10.3390/antiox13020231 (PMC10886084; doi:10.3390/antiox13020231)
Supplement: Supplementary file 1 [file antioxidants-13-00231-s001.zip › antioxidants-2802134-supplementary.pdf]

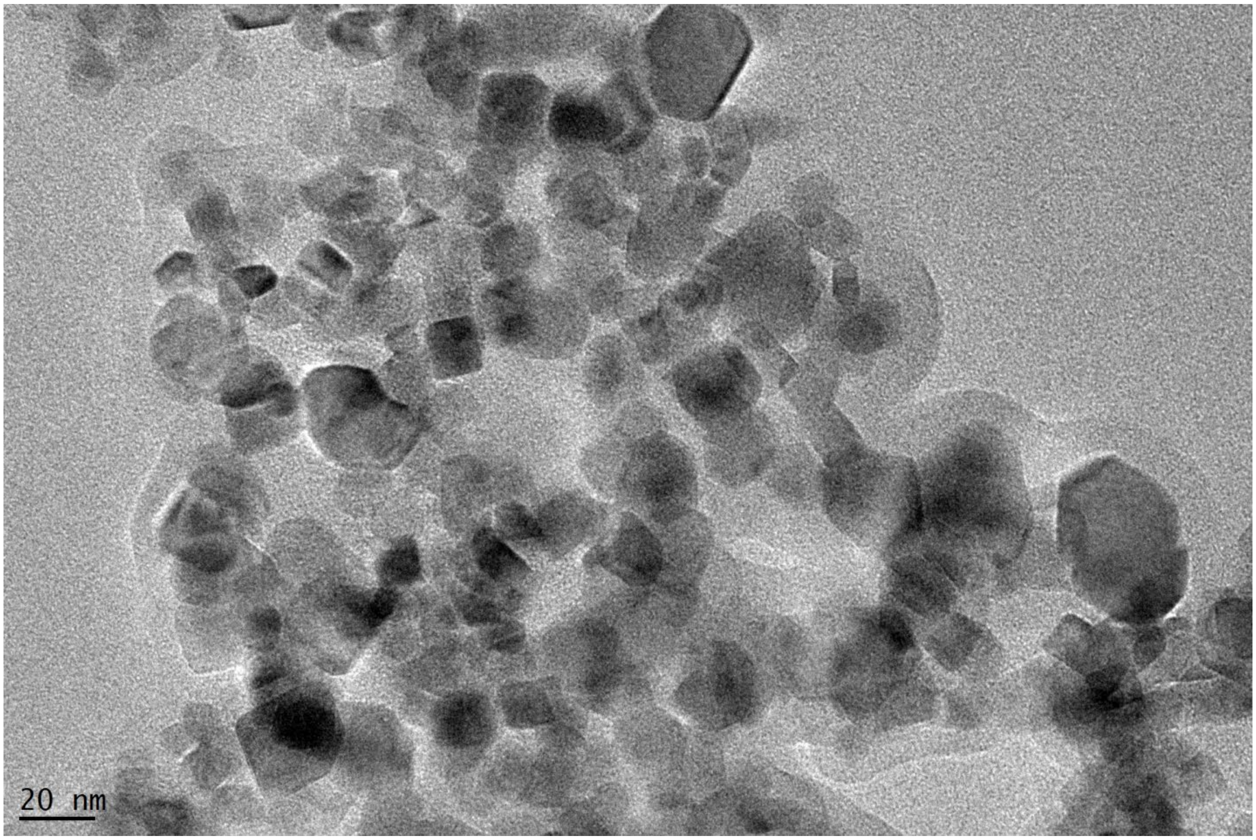

Figure S1. Typical TEM image of commercial TiO_2 nanoparticles (Degussa P25).

Besides oral acute treatment with vehicle (0.01 M HCl – a solution used to disperse NPs), we have additionally treated animals either with SA or 5-ASA (50 mg/kg), both dissolved in the vehicle.Systematic monitoring (average daily food and water consumption, total body mass gain/loss, regularity of estrus cycle, fractional contribution of organs of interest (ovaries, oviducts and uterus), hormone status and tumor marker levels, and levels of redox markers) was performed to detectpotential alterations in abovementioned parameters within all three experimental groupsduring animals' life and at the terminal experimental endpoint. Since SA and 5-ASA treated animals cycled in the same pattern as those in vehicle treated group, andalso, no variationsbetween the experimental groups were observed in the other investigated parameters, the SA and 5-ASA groups were excluded from further consideration.

Table S1. Average daily intake of food (g/day)and water (ml/day) and total body mass gain/loss (g) of young female rats after oral administration of vehicle, SA and 5-ASA treatment. C group - animals treated with vehicle (0.01 M HCl – a solution used to disperse NPs); SA group - animals treated with salicylic acid (SA); 5-ASA group - animals treated with 5-amino salicylic acid (5-ASA).

Group/parameter	C	SA	5-ASA
Food intake (g/day)	22.16 ± 0.42^{a}	22.50 ± 0.32^{a}	20.75 ± 0.48^{a}
Water intake (ml/day)	47.59 ± 0.85^{a}	50.00 ± 0.45^{a}	48.75 ± 0.57^{a}
Body mass gain/loss (g)	18.89 ± 2.47^{a}	20.00 ± 2.76^{a}	17.78 ± 3.45^{a}

Values with the same letter (a) did not differ significantly from each other ($p > 0.05$).

Table S2. Fractional contribution of ovaries, oviducts and uterus of young female rats 14 days after oral administration of of vehicle, SA and 5-ASA treatment. C group - animals treated with vehicle (0.01 M HCl – a solution used to disperse NPs); SA group - animals treated with salicylic acid (SA); 5-ASA group - animals treated with 5-amino salicylic acid (5-ASA).

Group/parameter	Body mass (g)	Wet ovaries mass/body mass (mg/g)	Wet oviducts and uterus mass/body mass (mg/g)
C	313.89 ± 4.06[a]	0.41 ± 0.02 [a]	1.98 ± 0.17[a]
SA	312.78 ± 5.47[a]	0.49 ± 0.04[a]	1.97 ± 0.05[a]
5-ASA	316.67 ± 4.79[a]	0.46 ± 0.04[a]	2.21 ± 0.12[a]

Values with the same letter (a) did not differ significantly from each other ($p > 0.05$).

Table S3. Hormone status and tumor marker levels of young female rats 14 days after oral administration of vehicle, SA and 5-ASA treatment. C group - animals treated with vehicle (0.01 M HCl – a solution used to disperse NPs); SA group - animals treated with salicylic acid (SA); 5-ASA group - animals treated with 5-amino salicylic acid (5-ASA).

Group/Hormone	C	SA	5-ASA
Estrogen (pmol/L)	132.11 ± 23.80 [a]	135.00 ± 10.21[a]	137.89 ± 13.49[a]
Progesterone (nmol/L)	112.77 ± 15.02 [a]	111.84 ± 8.39[a]	117.59 ± 13.55[a]
Testosterone (nmol/dL)	0.32 ± 0.11 [a]	0.34 ± 0.13[a]	0.33 ± 0.10[a]
Sex hormone-binding globulin (nmol/L)	<4.5	<4.5	<4.5
Follicle-stimulating hormone (IU/L)	<0.11	<0.11	<0.11
Luteinizing hormone (IU/L)	<0.12	<0.12	<0.12
Prolactin (mIU/L)	<17.22	<17.22	<17.22
**Group/Tumor marker**	**C**	**SA**	**5-ASA**
HE4 (pmol/L)	<20	<20	<20
CA 125 (U/mL)	<1.1	<1.1	<1.1

Values with the same letter (a) did not differ significantly from each other ($p > 0.05$).

Table S4. Levels of redox markers in ovaries of young female rats 14 days after oral administration of vehicle, SA and 5-ASA treatment. C group - animals treated with vehicle (0.01 M HCl – a solution used to disperse NPs); SA group - animals treated with salicylic acid (SA); 5-ASA group - animals treated with 5-amino salicylic acid (5-ASA).

Group/parameter	C	SA	5-ASA
PAB (HKU)	220.22 ± 7.59 [a]	219.22 ± 9.87[a]	217.35 ± 9.85[a]
AOPP (μmol/l)	92.48 ± 7.96 [a]	92.48 ± 9.71[a]	80.89 ± 8.81[a]
MDA (μM)	0.11 ± 0.00 [a]	0.09 ± 0.02[a]	0.10 ± 0.01 [a]
HNE (μM)	0.25 ± 0.02 [a]	0.27 ± 0.02[a]	0.27 ± 0.05[a]
MnSOD (U/mg)	4.97 ± 0.58 [a]	4.20 ± 0.24[a]	4.92 ± 0.44[a]
CuZnSOD (U/mg)	55.61 ± 5.60 [a]	49.94 ± 4.34 [a]	51.40 ± 1.59[a]
CAT (U/mg)	51.10 ± 5.11 [a]	48.71 ± 2.87 [a]	55.97 ± 3.08[a]
GPx (U/g)	20.03 ± 1.28 [a]	23.81 ± 2.06[a]	21.56 ± 1.30[a]
GSH/GSSG	0.70 ± 0.06 [a]	0.61 ± 0.13[a]	0.76 ± 0.04[a]
PC/LPC	1.60 ± 0.21 [a]	1.40 ± 0.17[a]	1.52 ± 0.12[a]

Values with the same letter (a) did not differ significantly from each other ($p > 0.05$).

Table S5. Levels of redox markers in uteri of young female rats 14 days after oral administration of vehicle, SA and 5-ASA treatment. C group - animals treated with vehicle (0.01 M HCl – a solution used to disperse NPs); SA group - animals treated with salicylic acid (SA); 5-ASA group - animals treated with 5-amino salicylic acid (5-ASA).

Group/parameter	C	SA	5-ASA
PAB (HKU)	318.74 ± 10.60 [a]	301.16 ± 14.33[a]	321.48 ± 11.60 [a]
AOPP (μmol/l)	62.38 ± 1.53 [a]	60.36 ± 6.73[a]	66.46 ± 9.31[a]
MDA (μM)	0.12 ± 0.01 [a]	0.13 ± 0.01 [a]	0.13 ± 0.01 [a]
HNE (μM)	0.25 ± 0.03 [a]	0.27 ± 0.03 [a]	0.28 ± 0.02[a]
MnSOD (U/mg)	5.35 ± 0.53 [a]	5.22 ± 0.59[a]	5.05 ± 0.68[a]
CuZnSOD (U/mg)	36.54 ± 2.66 [a]	37.29 ± 2.83 [a]	33.59 ± 3.29 [a]
CAT (U/mg)	59.36 ± 4.18 [a]	59.52 ± 2.25 [a]	60.23 ± 4.39 [a]
GPx (U/g)	33.37 ± 2.74 [a]	29.24 ± 1.75[a]	35.80 ± 3.14[a]
GSH/GSSG	0.87 ± 0.03 [a]	0.77 ± 0.08[a]	0.83 ± 0.03 [a]
PC/LPC	1.59 ± 0.10 [a]	1.56 ± 0.07[a]	1.49 ± 0.09[a]

Values with the same letter (a) did not differ significantly from each other ($p > 0.05$).

Table S6. Levels of redox markers in oviducts of young female rats 14 days after oral administration of vehicle, SA and 5-ASA treatment. C group - animals treated with vehicle (0.01 M HCl – a solution used to dissolve NPs); SA group - animals treated with salicylic acid (SA); 5-ASA group - animals treated with 5-amino salicylic acid (5-ASA).

Group/parameter	C	SA	5-ASA
PAB (HKU)	148.72 ± 11.63 [a]	163.62 ± 9.89[a]	143.84 ± 7.72[a]
AOPP (μmol/l)	65.19 ± 4.65 [a]	73.44 ± 5.44[a]	63.35 ± 3.59[a]
MDA (μM)	0.15 ± 0.01 [a]	0.15 ± 0.01 [a]	0.16 ± 0.01 [a]
HNE (μM)	0.24 ± 0.04 [a]	0.29 ± 0.02[a]	0.24 ± 0.06 [a]
MnSOD (U/mg)	5.00 ± 0.67 [a]	5.16 ± 0.51 [a]	5.14 ± 0.28[a]
CuZnSOD (U/mg)	26.79 ± 2.71 [a]	27.04 ± 2.83[a]	27.30 ± 2.73[a]
CAT (U/mg)	43.30 ± 4.04 [a]	44.15 ± 2.96 [a]	40.61 ± 2.59 [a]
GPx (U/g)	23.80 ± 2.87 [a]	23.34 ± 2.30[a]	25.24 ± 3.06 [a]
GSH/GSSG	1.49 ± 0.10 [a]	1.28 ± 0.09[a]	1.31 ± 0.10 [a]
PC/LPC	1.45 ± 0.16 [a]	1.34 ± 0.12[a]	1.32 ± 0.08[a]

Values with the same letter (a) did not differ significantly from each other ($p > 0.05$).
